# The research frontier of cesarean section recovery: A bibliometric analysis

**DOI:** 10.3389/fmed.2022.1071707

**Published:** 2022-12-13

**Authors:** Lizheng Zhao, Hong Wei

**Affiliations:** ^1^Department of Rehabilitation, Xiamen Humanity Rehabilitation Hospital, Xiamen, Fujian, China; ^2^Department of Rehabilitation Teaching and Research, Xi’an Siyuan University, Xi’an, China

**Keywords:** cesarean section (CS), recovery, enhanced recovery after surgery (ERAS), bibliometric analysis, Web of Science

## Abstract

**Background:**

Cesarean section (CS) has become an effective means to solve dystocia and some obstetric complications, and to save the lives of women and perinatal women. Disparities in quality obstetric care and rehabilitation in CS result from differences in health care systems across regions, and more scientific and reasonable rehabilitation programmes and management measures will benefit more parturient and newborns worldwide who must take CS. In this study, we performed a bibliometric analysis to collect a graphical representation of the CS recovery.

**Methods:**

A total of 995 documents of CS recovery were retrieved from the Web of Science Core Collection (WOSCC) on December 31, 2021, and then VOS viewer 1.6.18 was used for visual analysis.

**Results:**

Over the last 20 years, the researches of CS recovery have gradually increased and it will continue to grow in the next period. Anesthesia and Analgesia is the most popular journal in CS recovery. Most of the representative achievements are concentrated in the relevant institutions of European and American countries, Brendan Carvalho and Ian J. Wrench are among the outstanding scholars in this field, but the overall outcome is limited by limited regional work and lack of broad cooperation and representation. “CS,” “surgery,” “management,” “recovery,” “enhanced recovery,” and “risk factors” are high frequency keywords, and there is a close relationship between “management” and “enhanced recovery” around the CS and they also become one of the key factors to regulate the condition of patients.

**Conclusion:**

This work firstly analyzed the research condition of CS recovery by a bibliometric analysis. According to the practice guideline, it produces some outstanding representative productions, which involves enhanced recovery after surgery (ERAS) and will continue to be the focus of researchers. More substantive research articles and large-scale clinical studies may greatly enhance the scientific value, and it is necessary to strengthen the ERAS guideline and cooperation between researchers, generate broader consensus and results, and ultimately provide help for CS recovery.

## Background

Cesarean section (CS) is an important operation in obstetrics. Due to the advances in the knowledge of anesthesiology, blood transfusion, infusion, water and electricity balance, as well as the improvement of surgical methods, surgical suture materials and infection control measures, CS has become an effective means to solve dystocia and some obstetric complications, and to save the lives of women and perinatal women (1). Some data show that CS rates have increased by nearly 50% in the last 20 years (1, 2). In developed countries, CS rates are at their peak. In most developed countries, CS rates are around 30%, due to maternal factors such as advancing age and obesity, as well as medical developments that have made CS safer in terms of maternal and foetal morbidity and mortality (2, 3). In present-day obstetrics, cesarean delivery occurs in one in three women in the United States, and in up to four of five women in some regions of the world (3). CS is often considered a simple and safe alternative to natural delivery, but in some cases, it may be technically difficult and thus a health hazard for both mother and foetus (4, 5). As with any procedure, CS is associated with short – and long-term risks, particularly in Settings that lack the facilities or capacity to perform safe surgery or properly treat surgical complications, or where delivery care or repeat CS is not available as a matter of course in subsequent pregnancies (3, 4).

Surgery is a known physiological stress (6), in which preoperative preparation, operation and postoperative rehabilitation are important factors affecting the health of patients and children after cesarean section. Over the past 100 years, advances in CS technology have made it possible to reduce maternal morbidity and mortality. Nevertheless, maternal mortality and morbidity rates among women in developing countries and underdeveloped regions have increased significantly compared with those in developed regions (3). These disparities pose challenges to health care systems and represent inequalities in access to quality obstetric care and rehabilitation from CS. Therefore, to cope with the inequality of medical resources, it is particularly important to carry out rehabilitation work after cesarean section, involving uterine rehabilitation, physical recovery, pelvic floor muscle rehabilitation, scar management, breastfeeding and lactation function, etc. Physical therapy programs in the early stages of CS are effective and valuable for improving the quality and productivity of postnatal care, thereby improving post-delivery well-being and including reducing the amount of medication needed for pain control and improving the recovery of bowel activity (7). The implementation of a protocol of enhanced recovery for elective CS in a level III maternity is application safe and postoperative pain, nausea and vomiting are well managed, which has been involved in reducing adverse outcomes that can slow recovery, resulting in early discharge of patients while maintaining high levels of satisfaction (8). The application of rapid rehabilitation model of multidisciplinary cooperation and traditional Chinese medicine in CS can effectively improve the recovery rate, ensure the analgesic effect, and improve the maternal and infant outcomes, and has higher health and economic benefits, which is worthy of promotion (9, 10). Thus, more scientific and reasonable recovery programmes and management measures will benefit more parturient and newborns worldwide who must take CS.

In this study, we conducted a bibliometric analysis to gather a diagrammatic drawing of CS rehabilitation. Bibliometrics uses public academic literature data to analyze and track the progress of scientific data, reveal the structure of research and its productivity, evaluate the current status and trends of research, and predict the research prospects of a given topic (11, 12). The data will attract the interest and attention of researchers and enterprises in obstetrical department and parturient.

## Materials and methods

### Study selection

We retrieved all literature data regarding the caesarean section rehabilitation indexed in the Web of Science Core Collection (WOSCC). The term of caesarean section and rehabilitation were detected with MeSH. The documents from 2000 to 2021 (December 31, 2021) were searched, the language type was set to English, and the document type was set to Article and Review. The execution date of strategies was September 10, 2022 and the search terms and strategies used for the WOSCC database are as follow: #1, “Cesarean delivery” OR “Cesarean deliveries” OR “Cesarean section” OR “Caesarean section” OR “Abdominal delivery” OR “Abdominal deliveries” OR “Postcesarean section”; # 2, “Rehabilitation” OR “Recovery” OR “Physical medicine” OR “Physical therapy” OR “Occupational therapy”; # 3, “# 1” AND “# 2”; #4, #3 AND “Article and Review” AND “English.”

### Data collection

A total of 995 documents were retrieved from WOSCC database, and then the documents were used to make visual analysis ultimately. The title, publication year, authors, country, institution, keywords, journal, citation frequency, and relative citation ratio were analyzed. The 2021 impact factor (IF) of the journals were obtained from the Journal Citation Reports on September 15, 2022.

### Statistical analysis

To extract the most common topics, impactful authors and institutions, we chose the keywords and key references and the visualization of collaboration networks were conducted using VOS viewer version 1.6.8 (Leiden University, Leiden, Netherlands). We choose the keywords and key references to predict the research prospect and research hotspot. Keywords and key references were analyzed by VOS viewer. The parameters of the VOS viewer were set as follows: Method (Linlog/modularity).

## Results

### Publication outputs

**FIGURE 1:**
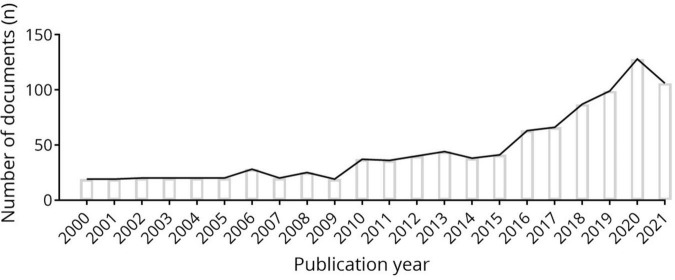
Annual number of documents indexed in the WOSCC from 2000 to 2021 by the online bibliometric analysis.

There were 995 documents of CS recovery from WOSCC databases database and which were used to make visual analysis ultimately. The count of annual publications from 2000 to 2021 was shown in Figure 1. It is with weak changes from 2000 to 2009, but the overall trend has gradually increased in recent years and it will continue to grow in the next period.

### Countries/regions and organization

**FIGURE 2:**
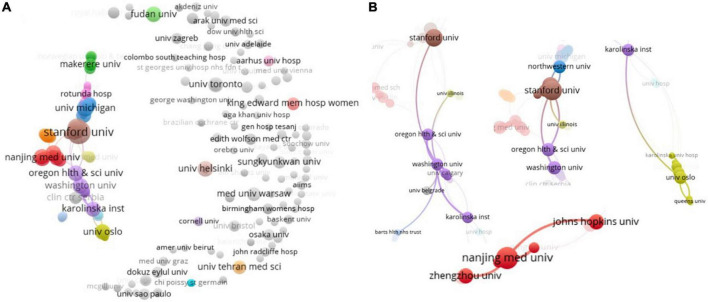
Co-author analysis of organizations with network visualization. **(A)** The largest set of 308 valid items were interlinked. **(B)** Some partners of organizations were performed.

A total of 76 countries/regions and 1,489 organizations participate in 995 productions were analyzed. As shown in Table 1, USA (*n* = 225) is the most productive countries and is well ahead of other countries. China, UK, Turkey, Australia, Canada, India, Iran, Germany, and Japan are the other productive countries of the top 10 institutions (Table 2). The citations of USA (*n* = 4,940) and UK (*n* = 2,997) are ahead, and UK leads China in both citations and total link strength (Table 2). Table 3 showed the top 10 institutions in terms of publications, mainly come from USA (*n* = 6), China (*n* = 2), Norway (*n* = 1), and Finland (*n* = 1). The top 4 ranked items by publications are Stanford University (*n* = 18), Duke University (*n* = 12), University of Michigan (*n* = 9), and Nanjing Medicinal University (*n* = 9), and the top 4 institutions by citations are Oregon Health and Science University (*n* = 408), Oslo University (*n* = 355), Washington University (*n* = 336), and Stanford University (*n* = 314), but Chinese institutions by publications and citations don’t attract much attention (Table 3). According to the statistical analysis, some of the publications are completed in cooperation with multiple institutions and they have cooperation with other institutions (Figure 2). In the network, the largest set of connected items consists of 308 items (Figure 2A). Most of institutions are isolated on the right side, including Fudan University and University of Helsinki. In contrast, Oregon Health and Science University, Washington University, Stanford University, and Oslo University have a wide range of partners (Figure 2B). Although Nanjing Medicinal University has low link strength (Table 3), they have a few good companions (including Zhengzhou University, Johns Hopkins University, etc., Figure 2B).

**TABLE 1:** Top 10 the most productive organizations.

Rank	Organizations	Country	Documents	Citations	Total link strength
1	Stanford University	USA	18	314	19
2	Duke University	USA	12	111	14
3	University of Michigan	USA	9	131	8
4	Nanjing Medicinal University	China	9	41	5
5	Oregon Health and Science University	USA	8	408	28
6	Washington University	USA	8	336	28
7	Oslo University	Norway	8	355	18
8	University of Helsinki	Finland	8	216	6
9	Harvard Medicinal University	USA	8	91	9
10	Fudan University	China	8	90	7

**TABLE 2:** Top 10 countries/regions on cesarean section recovery.

Rank	Country/Region	Documents	Citations	Total link strength
1	USA	225	4,940	62
2	China	136	1,178	7
3	UK	96	2,997	72
4	Turkey	65	734	6
5	Australia	38	801	19
6	Canada	36	895	43
7	India	34	246	3
8	Iran	32	296	1
9	Germany	31	1,118	30
10	Japan	31	362	3

**TABLE 3:** Top 10 with the largest number of publications.

Rank	Journals	Documents	2021 impact factor	2021 JCR partition
1	International Journal of Obstetric Anesthesia	44	3.282	Q2
2	Anesthesia and Analgesia	26	6.627	Q1
3	Medicine	21	10.871	Q3
4	Journal of Maternal Fetal Neonatal Medicine	20	2.323	Q3
5	Journal of Obstetrics and Gynaecology Research	20	1.697	Q4
6	European Journal of Obstetrics Gynecology and Reproductive Biology	16	2.831	Q3
7	BMC Pregnancy and Childbirth	15	3.105	Q2
8	Obstetrics and Gynecology	15	7.623	Q1
9	American Journal of Obstetrics and Gynecology	13	10.693	Q1
10	Regional Anesthesia and Pain Medicine	13	5.564	Q2

### Journals analysis

In total, 415 journals published research documents related to CS recovery from 2020 to 2021. In Table 4, the top 10 journals are shown that published about 20.40% of documents (203/995). *International Journal of Obstetric Anesthesia* is the most dynamic journal of CS recovery, followed by *Anesthesia and Analgesia*, *Medicine*, *Journal of Maternal Fetal Neonatal Medicine*, *Journal of Obstetrics and Gynaecology Research*, *European Journal of Obstetrics Gynecology and Reproductive Biology*, *BMC Pregnancy and Childbirth*, *Obstetrics and Gynecology*, *American Journal of Obstetrics and Gynecology*, and *Regional Anesthesia and Pain Medicine*. The IF of 10 journals was from 1.697 to 10.693, there are three journal citation reports (JCR) Q1 journals, and *American Journal of Obstetrics and Gynecology* shows the maximum IF of 10.693 (Q1), and *Obstetric Anesthesia* is with IF 3.282 and JCR Q2 (Table 3). According to the documents, IF and JCR partition, *Anesthesia and Analgesia* may be the most popular journal in CS recovery.

**TABLE 4:** Top 10 active authors with most documents.

Authors	Organizations	Country	Documents	Citations
Brendan Carvalho	Stanford University	USA	17	313
Ian J. Wrench	University of Sheffield	UK	7	436
Pervez Sultan	University College London Hospital	UK	7	104
Aaron B. Caughey	Oregon Health and Science University	USA	5	327
Gregg Nelson	University of Calgary	Canada	5	299
R. Douglas Wilson	Oregon Health and Science University	USA	5	299
Ashraf S. Habib	University of Minnesota	USA	5	57
Jeffrey Huang	University of Central Florida	USA	4	298
George A. Macones	Washington University	USA	4	303
Carol A. Aschenbrenner	Wake Forest School of Medicine	USA	4	38

### Authors analysis

**FIGURE 3:**
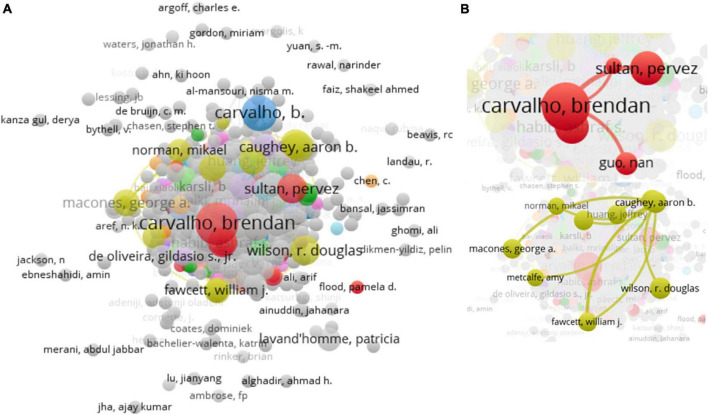
Co-occurrence analysis of authors. **(A)** 4,934 items valid items were connected. **(B)** The cooperation of Brendan Carvalho and Aaron B. Caughey were presented.

A total of 4,934 authors drafted the 995 documents in CS recovery. In Table 4, the first three most active authors are from the Stanford University (USA), University of Sheffield (UK), and University College London Hospital (UK), Brendan Carvalho is the most active author in CS recovery (with 17 documents and 313 citations), and Ian J. Wrench (University of Sheffield) is the highest citation researcher. Subsequent authors have similar production, but the citations of Ashraf S. Habib and Carol A. Aschenbrenner are weaker (in Table 4). The co-authorship map of all authors was generated (4,934 items, Figure 3A). The connection between authors is loose and most scholars are scattered independently with other activated researchers (Figure 3A). As shown in Figure 3B, the partners of Brendan Carvalho and Aaron B. Caughey are relatively simple and the lack more extensive contacts.

### Citation analysis

**FIGURE 4:**
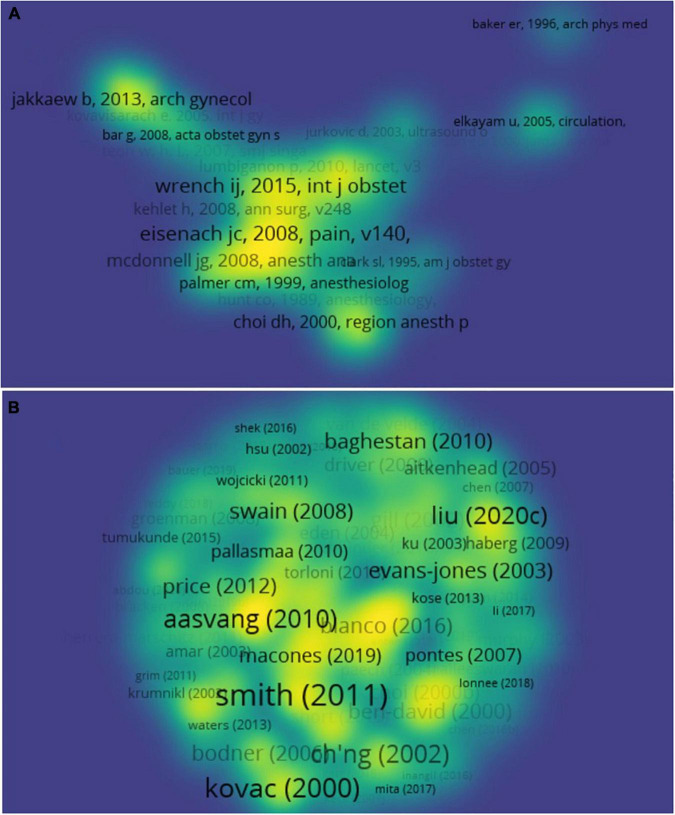
Citation analysis of documents. **(A)** The co-citation map of 1,272 cited references was generated. **(B)** The citation map of documents.

Of the 23,261 cited reference, 1,272 meet the threshold (minimum number of documents of an author: 3) and the co-citation map of cited references was generated (Figure 4A). “Severity of acute pain after childbirth, but not type of delivery, predicts persistent pain and postpartum depression” (13) is the highest cited reference of CS recovery (with 31 citations; Table 5), and it is also the most visible center of the network (Figure 4A). As the practice guideline, “Guidelines for Antenatal and Preoperative care in Cesarean Delivery: Enhanced Recovery After Surgery Society Recommendations (Part 1),” “Guidelines for intraoperative care in cesarean delivery: Enhanced Recovery After Surgery Society Recommendations (Part 2),” and “Guidelines for postoperative care in cesarean delivery: Enhanced Recovery After Surgery (ERAS) Society recommendations (part 3)” are also widely noted, recognized, and cited. In this field, it also produces some outstanding representative productions (Table 6). The top 3 citations of documents are “Perioperative fasting in adults and children: guidelines from the European Society of Anaesthesiology” (review), “Prevention and treatment of postoperative nausea and vomiting” (review), and “Predictive risk factors for persistent postherniotomy pain,” and they are also the visible center of the network (article; Table 6 and Figure 4B). The data suggest Ian Smith is very interested in the research of CS recovery, but the researches of Eske K. Aasvang is more important drivers in the development of the field.

**TABLE 5:** Top 10 Co-citation of cited reference.

Rank	Production	First author	Source	Type	Publication year	Total citations
1	Severity of acute pain after childbirth, but not type of delivery, predicts persistent pain and postpartum depression	James C. Eisenach	Pain	Article	2008	31
2	Introduction of enhanced recovery for elective caesarean section enabling next day discharge: a tertiary centre experience	I. J. Wrench	Int J Obstet Anesth	Article	2015	30
3	Guidelines for postoperative care in cesarean delivery: Enhanced Recovery After Surgery (ERAS) Society recommendations (part 3)	George A. Macones	Am J Obstet Gynecol	Review	2019	28
4	Guidelines for Antenatal and Preoperative care in Cesarean Delivery: Enhanced Recovery After Surgery Society Recommendations (Part 1)	R. Douglas Wilson	Am J Obstet Gynecol	Review	2018	28
5	Guidelines for intraoperative care in cesarean delivery: Enhanced Recovery After Surgery Society Recommendations (Part 2)	Aaron B. Caughey	Am J Obstet Gynecol	Review	2018	26
6	The analgesic efficacy of transversus abdominis plane block after cesarean delivery: a randomized controlled trial	John G. McDonnell	Anesth Analg	Article	2008	22
7	Enhanced recovery after elective caesarean: a rapid review of clinical protocols, and an umbrella review of systematic reviews	Ellena Corso	BMC Pregnancy Childbirth	Review	2017	21
8	Effects of gum chewing on recovery of bowel function following cesarean section: a randomized controlled trial	Bordin Jakkaew	Arch Gynecol Obstet	Article	2013	21
9	Intraoperative and postoperative analgesic efficacy and adverse effects of intrathecal opioids in patients undergoing cesarean section with spinal anesthesia: a qualitative and quantitative systematic review of randomized controlled trials	J. B. Dahl	Anesthesiology	Article	1999	21
10	Enhanced recovery from obstetric surgery: a U.K. survey of practice	S. Aluri	Int J Obstet Anesth	Article	2014	20

**TABLE 6:** Top 10 citation analysis of documents.

Rank	Title	First author	Source	Type	Publication year	Total citations
1	Perioperative fasting in adults and children: guidelines from the European Society of Anaesthesiology	Ian Smith	Eur J Anaesthesiol	Review	2011	447
2	Prevention and treatment of postoperative nausea and vomiting	A. L. Kovac	Drugs	Review	2000	317
3	Predictive risk factors for persistent postherniotomy pain	Eske K. Aasvang	Anesthesiology	Article	2010	240
4	Prospective study of liver dysfunction in pregnancy in Southwest Wales	C. L. Ch’ng	Gut	Article	2002	232
5	Pregnancy and Perinatal Outcomes of Women With Coronavirus Disease (COVID-19) Pneumonia: A Preliminary Analysis	Dehan Liu	AJR Am J Roentgenol	Article	2020	219
6	Multifactorial preoperative predictors for postcesarean section pain and analgesic requirement	Peter H. Pan	Anesthesiology	Article	2006	181
7	Quadratus Lumborum Block Versus Transversus Abdominis Plane Block for Postoperative Pain After Cesarean Delivery: A Randomized Controlled Trial	Rafael Blanco	Reg Anesth Pain Med	Article	2016	170
8	Catastrophizing: a predictive factor for postoperative pain	Am J. Surg	Am J Surg	Review	2011	165
9	Balloon-assisted occlusion of the internal iliac arteries in patients with placenta accreta/percreta	Leonard J. Bodner	Cardiovasc Intervent Radiol	Article	2006	151
10	Congenital brachial palsy: incidence, causes, and outcome in the United Kingdom and Republic of Ireland	G. Evans-Jones	Arch Dis Child Fetal Neonatal Ed	Article	2003	147

### Keywords analysis

**FIGURE 5:**
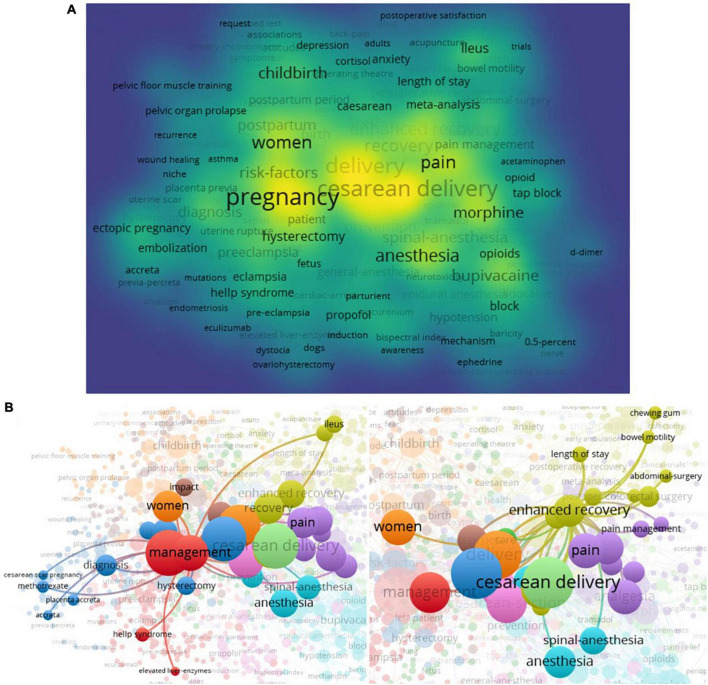
Co-occurrence analysis of keywords. **(A)** The network visualization of total keywords (1,004 keywords) was conducted. **(B)** The crosstalk of keywords “management” and “enhanced recovery” were presented.

Of the 3,968 keywords, 1,004 meet the threshold (minimum number of documents of a keyword: 2) and the co-occurrence map of keywords was generated (Figure 5). “CS,” “surgery,” “management,” “recovery,” “enhanced recovery,” and “risk factors” are high frequency keywords and are also given highlights in the relationship network (Figure 5A). Further analysis reveals a close relationship between “management” and “enhanced recovery” around the CS (Figure 5B), and they also become one of the key factors to regulate the condition of patients.

## Discussion

Over the last 20 years, the researches of CS recovery have gradually increased and it will continue to grow in the next period. Anesthesia and Analgesia is the most popular journal in CS recovery. Most of the representative achievements are concentrated in the relevant institutions of European and American countries, Brendan Carvalho and Ian J. Wrench are among the outstanding scholars in this field, but the overall outcome is limited by limited regional work and lack of broad cooperation and representation. According to the practice guideline, it produces some outstanding representative productions, which involves management and enhanced recovery.

Vaginal birth is a natural physiological process, but CS may be necessary to protect the health of the woman and the baby in certain circumstances. In these cases, underuse of CS leads to increased maternal and perinatal mortality and morbidity (14). In contrast, overuse does not show benefits, but may cause harm and waste human and financial resources. On the other hand, failure to obtain a CS in a timely manner can lead to perinatal asphyxia, stillbirth, uterine rupture or obstetric fistula, which is a sign of unusually long obstructed labor (15). Thus, CS should be approached carefully in its testify and aim to make reproductive have services accessible to all parturient who need them (15). Optimizing CS use and rehabilitation management is a global concern and public health challenge (4).

Scientific management after CS will be beneficial to rehabilitation. (1) Postoperative monitoring immediately after delivery should be performed in the recovery room, but in special circumstances, it may be performed in the delivery unit, and provided safety rules are maintained and regulators are notified that specific surveillance, including emergency call procedures, must be carried out (16). (2) Systematic blood count immediately after CS is not recommended in the general population. (3) The analgesic regimen developed by the medical team should be appropriate for each patient, and early feeding and drinking under regional or general anesthesia is recommended after elective or emergency CS (17, 18). (4) Indwelling bladder catheter should be inserted before operation and maintained during operation; The bladder catheter should be removed preferentially within 12 h after CS; It is recommended to check for spontaneous urination within 4–6 h after removal of the bladder catheter. If the patient fails to empty within 6 h of extubation, the attending physician should be notified (19–21). (5) Patients are advised and encouraged to get out of bed as early as day 1 (or 6–8 h). (6) Prophylactic treatment with two antiemetics is recommended during CS (22). (7) Whether to add low molecular weight heparin, for obese patients, the dose of low molecular weight heparin needs to be appropriate to body weight (23, 24). (8) Anti-embolic Stockings are recommended for thrombosis prevention on the morning of surgery and for at least 7 days after surgery (16, 25).

ERAS programs are standardized perioperative care plans that, when combined with an audit system and a dedicated multidisciplinary team, can reduce surgical stress, enhance physiological and functional recovery, reduce length of stay, and reduce complications (26, 27). There is increasing evidence to support the success of ERAS for a wide range of surgical procedures, including colorectal, urology, gynecology, and hepatobiliary surgery (27, 28). Then the ERAS principles in obstetrical surgery are also being tried (6, 29). Teigen et al. conduct a randomized controlled trial and reveal that ERAS at CS presents the potential to improve outcomes such as day of discharge is suggested by the observed reduction in overall postoperative length of stay, improved patient satisfaction, and an increase in breastfeeding rates (30). ERAS also plays an active role in improvement of postoperative pain, intraoperative nausea, hospitalization cost, and patient satisfaction. In a prospective randomized controlled trial, Pan et al. find the ERAS group has significantly fewer patients with intraoperative nausea, pain of visual analog scale (VAS) scores, and VAS grade >3 during rest in the first 24 h and during motion in the first 24 and 48 h after CS; And patient satisfaction rated as per the VAS was significantly higher in the ERAS group (31).

Preliminary studies on the implementation of ERAS after CS have been carried out mainly outside Europe, in which early oral feeding, early mobilization and timely removal of catheter are important components of ERAS, which are mainly performed in patients undergoing scheduled CS (29). In 2017, the ERAS Social Guidelines Committee selected an expert group to review and prepare guidelines for perioperative care of CS. Based on the available evidence in 2017, the recommendations were published in 2018–2019 and are divided into three parts: antenatal and preoperative care, intraoperative care, and postoperative care (32–34). In 2019, the Society of Obstetrical Anaesthesia and Perinatology (SOAP) compiled a consensus document regarding ERAC, which provides recommendations from SOAP on the elements that should be included in ERAS paths, including basic core elements and other recommended elements (17). For the postoperative care, elements include as follow: (1) offer ice chips and water within 1 h postoperatively, consider gum chewing (gum chewing starting right after CS three times a day for about 30 min until the first flatus (35); (2) advance to regular diet within 4 h postoperatively; (3) heparin/saline lock IV once oxytocin infusion complete and tolerating fluids (36); (4) maintain normoglycemia with <180–200 mg/dl; (5) minimize opioid consumption and continue scheduled nonopioid analgesia (37); (6) ambulation should occur soon after motor function returns, beginning with dangling and out of bed to chair and progressively increasing to 3–4 times after postoperative day 1; (7) removal of urinary catheter 6–12 h postoperatively (early removal of the indwelling urinary catheter in patients who underwent elective CS showed significant less dysuria, less urinary frequency and a decrease in the incidence of significant bacteriuria (20); (8) provide early and robust lactation support (early breastfeeding, adequate sucking stimulation, proper sucking technique, and limited formula may be effective in improving long-term breastfeeding for mothers who have delivered by cesarean section (38); (9) coordinate and streamline discharge processes to facilitate early discharge; (10) limit unnecessary interruptions to maximize rest and bonding. The latest expert consensus also stresses the core outcome, which include compliance with enhanced recovery protocol; fasting times; times to mobilization and urinary catheter removal; provision of optimal analgesia (maternity satisfaction, compliance with analgesia, opioid consumption or requirement and incidence of nausea or vomiting); early breastfeeding success; length of hospital stay; and hospital re-admissions (39). And the outcomes should be considered when designing future enhanced recovery studies.

Due to differences in economic, medical and educational levels, the recent data show CS rates are more than 15% in 63% countries but lower than 10 in 28% countries (1, 40). So, it is important that patients must be educated about the risks of cesarean section as part of pregnancy education, and providers must consider the long-term risks when deciding whether to perform a cesarean section. Educating women about the potential short – and long-term risks of cesarean section to mother and baby is critical to the success of this mission and will also contribute to the medical compliance and effectiveness of rehabilitation management after cesarean section. For the future, the mission continues as we pursue twenty-first century solutions to address alarming rates of obstetric bleeding, perinatal hysterectomies, maternal mortality and unequal resources in health care (3).

## Limitations

Some limitations should be addressed in this work. Firstly, the deadline for researched publications was December 31, 2021, but WOSCC would also keep updating, many documents are still being updated in 2022. Besides, the terms of “Cesarean delivery,” “Cesarean deliveries,” “Cesarean section,” “Caesarean section,” “Abdominal delivery,” “Abdominal deliveries,” “Postcesarean section,” “Rehabilitation,” “Recovery,” “Physical medicine,” “Physical therapy,” “Occupational therapy,” “English,” “Article” and “Revies” were selected to define the topic of the studies, not all documents were completely obtained, such as the Meeting, Case Report, Clinical Trial, Patent and other multiple document types. Thirdly, because the search was limited to WOS Core Collection databases, some documents MEDLINE^®^, KCI-Korean Journal Database, and SciELO Citation Index were missed. However, we believe that the overall situation and general trend of these analyses are consistent with the research blueprints of CS recovery.

## Conclusion

This work firstly analyzed the research condition of CS recovery by a bibliometric analysis. The data showed CS recovery may be an interesting field of research, but the output and cooperation of more representative works still need to be improved. According to the practice guideline, it produces some outstanding representative productions, which involves ERAS and will continue to be the focus of researchers. More substantive research articles and large-scale clinical studies may greatly enhance the scientific value, and it is necessary to strengthen the ERAS guideline and cooperation between researchers, generate broader consensus and results, and ultimately provide help for CS recovery.

## Data availability statement

The original contributions presented in this study are included in the article/supplementary material, further inquiries can be directed to the corresponding author.

## Author contributions

HW: conceptualization and writing – review and editing. LZ: data collection and analysis and writing – original draft. Both authors contributed to the article and approved the submitted version.
